# Signalling interaction between β‐catenin and other signalling molecules during osteoarthritis development

**DOI:** 10.1111/cpr.13600

**Published:** 2024-01-10

**Authors:** Jing Feng, Qing Zhang, Feifei Pu, Zhenglin Zhu, Ke Lu, William W. Lu, Liping Tong, Huan Yu, Di Chen

**Affiliations:** ^1^ Department of Orthopedics, Traditional Chinese and Western Medicine Hospital of Wuhan Tongji Medical College, Huazhong University of Science and Technology Wuhan Hubei China; ^2^ Department of Orthopedics Wuhan No. 1 Hospital Wuhan Hubei China; ^3^ Department of Emergency Renmin Hospital, Wuhan University Wuhan Hubei China; ^4^ Department of Orthopedic Surgery the First Affiliated Hospital of Chongqing Medical University Chongqing China; ^5^ Faculty of Pharmaceutical Sciences Shenzhen Institute of Advanced Technology Shenzhen China; ^6^ Research Center for Computer‐aided Drug Discovery Shenzhen Institute of Advanced Technology, Chinese Academy of Sciences Shenzhen China

## Abstract

Osteoarthritis (OA) is the most prevalent disorder of synovial joint affecting multiple joints. In the past decade, we have witnessed conceptual switch of OA pathogenesis from a ‘wear and tear’ disease to a disease affecting entire joint. Extensive studies have been conducted to understand the underlying mechanisms of OA using genetic mouse models and ex vivo joint tissues derived from individuals with OA. These studies revealed that multiple signalling pathways are involved in OA development, including the canonical Wnt/β‐catenin signalling and its interaction with other signalling pathways, such as transforming growth factor β (TGF‐β), bone morphogenic protein (BMP), Indian Hedgehog (Ihh), nuclear factor κB (NF‐κB), fibroblast growth factor (FGF), and Notch. The identification of signalling interaction and underlying mechanisms are currently underway and the specific molecule(s) and key signalling pathway(s) playing a decisive role in OA development need to be evaluated. This review will focus on recent progresses in understanding of the critical role of Wnt/β‐catenin signalling in OA pathogenesis and interaction of β‐catenin with other pathways, such as TGF‐β, BMP, Notch, Ihh, NF‐κB, and FGF. Understanding of these novel insights into the interaction of β‐catenin with other pathways and its integration into a complex gene regulatory network during OA development will help us identify the key signalling pathway of OA pathogenesis leading to the discovery of novel therapeutic strategies for OA intervention.

## Graphical abstract

The Wnt/β‐catenin signalling pathway plays critical roles in OA pathogenesis. Abnormal β‐catenin signalling in chondrocytes, synovial fibroblast and osteocytes leads to OA‐like structural changes, such as progressive cartilage lesion, synovitis and sclerosis of subchondral bone and osteophyte formation.

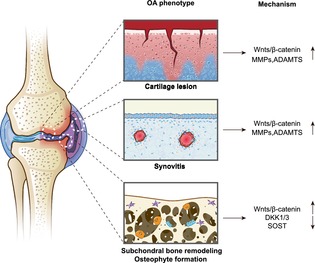

## 1 OSTEOARTHRITIS

Osteoarthritis (OA) is the most common disorder of synovial joint and prevalent musculoskeletal disease affecting middle‐aged and older individuals, poses a major challenge for public health care systems in 21st century.^1^, ^2^ A systematic analysis of the Global Burden of Disease Study in 2017 reported that the global prevalence of OA was 3.75% and annual incidence rate of OA was 1.81‰, an increase of 9.3% and 8.2% from 1990, respectively.^3^, ^4^ And the reported global age‐standardized Years Lived With Disability (YLD) rate was 118.8, an increase of 9.6% from 1990. In China, it is estimated that the prevalence rates of symptomatic OA of the knee is 8.1%, with a prevalence of 5.7% and 10.3% in men and women in 2012. In contrast, the prevalence rates of patients with symptomatic knee OA is 17%, with 12.3% in males and 22.2% in females in 2018, suggesting that larger population was affected in recent years and women are more susceptible to OA.^5^ With regard to the prevalence of knee OA in different geographical location, the rural areas and the southern China were much higher than that of urban regions and northern China.^6^ An epidemiological study was conducted to analyse the overall prevalence of OA in different joints and found that lumbar OA was the highest prevalent, followed by knee OA, and non‐weight‐bearing hand joint OA.^7^

Although OA has long been attributed to mechanical ‘wear and tear’, it is now recognized as a complex disease involving the whole joint pathology featured by cartilage break‐down, synovitis, abnormal remodelling of subchondral bone and marginal osteophyte outgrowth, leading to joint dysfunction, pain, stiffness, functional limitation.^8^, ^9^, ^10^ Aging, obesity, female gender, race, genetics, physical labour occupation, hypertension, abnormal joint strength lines and previous joint injury increase the risk of OA development.^10^, ^11^, ^12^

Current treatment options available for OA patients include oral nonsteroidal, anti‐inflammatory drugs (NSAIDs), acetaminophen and opioid analgesics, as well as intra‐articular injections, such as steroids, hyaluronic acids and platelet‐rich plasma. These treatment strategies are focused on relieving OA pain and improving joint function, none of them halt the disease progression.^10^, ^13^ Some of them even have substantial side effects or toxicity when investigated over a long period of time. Great effort has been devoted to evaluate OA pathogenesis, and contributions of varieties of intrinsic signalling pathways in joint degeneration have been identified such as Wnt/β‐catenin, Indian Hedgehog (Ihh), transforming growth factor β (TGF‐β), epidermal growth factor receptor (EGFR), bone morphogenic protein (BMP), fibroblast growth factor (FGF), nuclear factor κB (NF‐κB), and Runt‐related transcription factor 2 (Runx2), HIF, and Notch.^14^, ^15^, ^16^, ^17^ However, effective drugs targeting a specific pathway to halt cartilage degradation has not been generated. Patients with OA finally receive joint replacement surgery after suffering from joint pain and stiffness for long time, highlighting the importance and urging need of finding new effective strategies for OA treatment. Therefore, clarifying the pathogenesis of OA and then developing high‐efficacy and low‐toxicity anti‐OA drugs are essential. This review focuses on recent progress in the understanding of the importance of Wnt/β‐catenin underlying OA pathogenesis and the interplay between Wnt/β‐catenin and other signallings including TGF‐β, BMP, Ihh, NF‐κB, FGF, Runx2. Novel insights into the interaction of signalling network during OA development will help us identify the key signalling pathway in OA development, as well as define how Wnt/β‐catenin is integrated with other signalling pathways in a complex gene regulatory network.

## 2 WNT/Β‐CATENIN SIGNALLING OVERVIEW

As highly conserved signalling pathway, the Wnt/β‐catenin system is vital for morphogenesis and cell organization during embryo‐genesis.^18^, ^19^ Wnt is a combination of the wingless gene of Drosophila and integrase‐1 (Int1) gene in mouse breast cancer. Since Nusse and colleague identified the first member of the Wnt family in 1982, the Wnt/β‐catenin signalling pathway has been widely studied, and the evidence on its regulation and its function in health and disease gradually emerge.^20^, ^21^ Wnt signalling pathways were categorized into non‐canonical and canonical signalling pathways. The former are independent of β‐catenin, including Wnt‐Ca^2+^ signalling, Wnt‐planar cell polarity signalling and Wnt/lipoprotein receptor‐related protein 6 (LRP6) pathways, related to Wnt/stabilization of proteins signalling as well as Wnt/target of rapamycin (TOR) signalling, which are of importance in regulating cell polarity and migration. The latter is β‐catenin‐dependent signalling pathways comprises the following key elements: the extracellular Wnt ligands including Wnt3a and Wnt1, the membrane receptors like Frizzled proteins (FZD) receptors, LRP5 and LRP6, and destruction complex components, that is, adenomatosis polyposis coli (APC)/Axin/protein phosphatase 2A/glycogen synthase kinase 3 (GSK‐3)/casein kinase 1α (CK‐1α), dishevelled (DVL) in cytoplasm, as well as β‐catenin and T cell factor (TCF)‐lymphoid enhancer factor (LEF) transcription factors in nucleus.

In the absence of Wnt ligands, β‐catenin acts as an intercellular adhesion adaptor protein and transcriptional co‐regulator, which is restricted within a multiprotein complex of APC/Axin/GSK‐3β. Then β‐catenin is phosphorylated by GSK‐3 and CK1 and subsequently degraded by ubiquitin‐dependent proteolysis. In the presence of the Wnt ligands, Wnt ligands bind to the FZD receptor and its co‐receptor LRP5/LRP6. Next, phosphorylation of DVL results in GSK‐3β destruction complex dissociation, which mediates β‐catenin phosphorylation. Subsequently β‐catenin flows into nucleus location, and interacts with TCF/LEF, promoting the expression of Myc, Ccnd1, and Dkk1, etc.

## 3 Β‐CATENIN SIGNALLING IN OA CARTILAGE

Previous human studies have revealed that abnormal β‐catenin signalling correlated with OA progression.^22^ Markedly increased β‐catenin expression have been reported in OA cartilage, and β‐catenin overexpression in chondrocytes upregulate matrix degrading enzymes.^23^, ^24^ The importance of β‐catenin protein in the development of cartilage was found using conditional gene upregulation of β‐catenin in mouse cartilage. Activation of Wnt/β‐catenin causes rapid increased expression of matrix metalloproteinase (MMPs) and a disintegrin like and metalloproteinase with thrombospondin type I motif (Adamts), breaking down cartilage extracellular matrix such as aggrecan and proteoglycan.^25^ Our laboratory had first established conditional β‐catenin activation mice, β‐catenin(ex3)^Col2ER^, in which β‐catenin accumulated in chondrocytes due to inhibition of β‐catenin degradation.^26^ These mice developed OA‐like structural changes, such as progressive cartilage lesion, sclerosis of subchondral bone and osteophyte formation.^26^ Studies presented that β‐catenin expression correlated positively with OA severity in knee OA samples from patients. Furthermore, our previous data have found that activation of β‐catenin causes multiple joints OA such as knee joint, hip joint, temporomandibular joint and lumber facet joint.^26^, ^27^, ^28^, ^29^ Conversely, to explore the function of β‐catenin when β‐catenin signalling is specifically suppressed in chondrocytes, we generated *Col2‐ICAT* mice. These mice display delayed chondrocyte maturation in growth plate and decelerated articular cartilage erosion.^30^ Furthermore, Col2‐specific knockout of *β‐catenin* led to bone destruction in knee joint area, large numbers of osteoclasts and adipocytes were accumulated in the area underneath of growth plate.^31^ In a study using *Col2a1‐Wnt16* and *Col2a1‐Cre;Wnt16* ^*fl/fl*^ mice as well as 3D chondrocyte pellet culture with technology of adenovirus Wnt16 manipulation, Tong and colleagues reveal that Wnt16 inhibit chondrocyte hypertrophy via activating PCP/JNK and cross‐talking with mTORC1‐PTHrP pathway.^32^ Chen et al. demonstrated that cyclin D1 stimulated proliferation and apoptosis of the chondrocyte through increasing the expression of Wnt3/β‐catenin rather than Wnt10a/β‐catenin signalling pathway.^33^ In a study, *Prg4‐CreER;Ctnnb1* ^*fl/fl*^ and *Prg4‐CreER;Ctnnb1‐ex3* ^*fl/wt*^ mice were generated for β‐catenin loss‐ and gain‐of‐function, respectively. In this study, the authors found that Prg4 was highly and specifically expressed in the superficial areas of cartilage in adult mice. In superficial zone‐specific *β‐catenin* KO mice, the OA pathology was accelerated, whereas cartilage degeneration was ameliorated in superficial zone‐specific *β‐catenin* stabilized mice.^34^ Dietary magnesium deficiency accelerates OA development by decreasing chondrocytes autophagy through Wnt/β‐catenin signalling activation.^35^ These data indicate that Wnt/β‐catenin signalling contribute to the maintenance of the homeostasis of articular cartilage and play a vital role on OA pathogenesis.

## 4 Β‐CATENIN SIGNALLING IN OA SUBCHONDRAL BONE

The subchondral bone is defined as the space including subchondral bone plate and the underlying trabecular bone with a different biomechanical and biochemical characteristic from the overlying articular cartilage.^36^, ^37^, ^38^ It is well established that the classical Wnt pathway acts as a major regulator of osteogenesis in subchondral bone. The function of Wnt/β‐catenin in bone was first discovered by identifying mutated genes for rare diseases, such as osteoporosis and pseudoglioma syndrome. For example, LRP5, a co‐receptor of Wnt signalling, loss‐of‐function and gain‐of‐function mutations closely linked with osteoporosis/pseudoglioma syndrome and high bone mass syndrome, respectively.^39^, ^40^, ^41^ SOST is a gene that encodes Sclerostin and Sclerostin is an endogenous inhibitor of Wnt and was identified in another rare human diseases, sclerosis and Van Buchem disease.^42^, ^43^, ^44^ Later genome‐wide association studies (GWAS) further confirmed that the polymorphisms of LRP5, Axin1, SFRP4, R‐spondin 3, LRP4, Dkk1 and Wnt16 in the Wnt signalling pathway are closely related to variation of bone mineral density.^45^, ^46^, ^47^ These findings establish the vital role of the classical Wnt pathway as a key regulator of osteogenesis and an ideal therapeutic target.

Previous studies have found that Wnt activation in OA subchondral bone and osteophytes and Dkk‐1 decreased subchondral bone fraction and osteophyte volume.^48^ Moreover, a study demonstrated that increased β‐catenin expression but decreased DKK3 expression in osteocytes with arthritis development. This study shows an opposite roles of DKK3 and β‐catenin in OA subchondral bone remodelling. Further, it is reported that reduced SIRT1 expression and increased SOST levels were detected in OA osteoblast, which decreases Wnt/β‐catenin activity. Decreased SOST expression protected cartilage by inhibiting MMPs and Adamts, but promoted subchondral bone sclerosis. Recently, a study reported upregulated β‐catenin and downregulated SOST in hypoxic osteoblasts and increased serum levels of DKK‐1 in patients with OA. They concluded that hypoxia in subchondral bone contributes to the crosstalk between chondrocytes and osteoblasts and modifying chondrocytes toward an OA‐like phenotype by inducing the Wnt/β‐catenin activation in osteoblasts.^49^

The integrity of the human bone is maintained by continuous processes of osteoclast‐triggered bone resorption coupled with osteoblast‐mediated bone formation, known as ‘bone remodelling’, which are tightly controlled and precisely coordinated.^50^, ^51^ In the process of bone remodelling, receptor activator of NF‐κB ligand (RANKL) binds to RANK on osteoclast progenitors, stimulates NF‐κB activation to promote osteoclast differentiation. However, osteoprotegerin (OPG) can act as the receptor of RANKL and compete with RANK for binding RANKL, thus inhibiting the generation of osteoclasts. Considering relatively slower turnover rate of cartilage, subchondral bone went through more rapid and uncoupled modelling and remodelling process, in which increased bone resorption and bone formation exist at different OA stages in response to the changes of the mechanical microenvironment. These abnormal remodelling phenotypes of subchondral bone include osteophyte formation, thickening and sclerosis of the subchondral bone plate, and changes in trabecular bone micro‐structures as well as the development of subchondral bone cysts and bone marrow oedema, which are commonly observed at weight‐bearing zone of subchondral bone in OA patients.^52^ In early‐stage OA, osteocytes promote osteoclast differentiation by increasing RANKL:OPG ratio. However, in severe OA osteocytes enhance osteoblast mineralization through upregulating Wnt proteins and reducing SOST secretion.

Theoretically, the use of anti‐osteoclast drugs might have protective effects on subchondral bone, particularly in the phase of increased subchondral bone remodelling and hypomineralization.^53^ Indeed, clinical studies tend to support it. In a study, Bruyere and colleagues found that a 3‐year administration with strontium ranelate for patients with osteoporosis and spinal OA markedly reduce low‐back pain and the development of the radiographic spinal OA compared with placebo.^54^ Laslett et al performed clinical research and showed that a single intravenous injection of zoledronic acid significantly reduced VAS pain scores of knee and BML size after 6 months.^55^ The results of a randomized placebo‐controlled trial (RCT) evaluating efficacy and safety of oral administered strontium ranelate in the treatment of OA by Reginster et al. demonstrated that treatment with strontium ranelate for OA was associated with reduced joint space width and fewer radiological progressors over 3 years.^56^ Greater reductions in total WOMAC score, VAS pain subscore, physical function subscore and knee pain were observed in patients with administration of strontium ranelate 2 g/day. More recently, Hayes and colleagues concluded that bisphosphonate therapy may benefit for knee OA patients in early‐stage with non‐overweight rather than advanced stage or with overweight because it protect against OA radiographic progression. And upregulation of SOST and DKK‐1 expression but decreased total SOST concentrations were observed among women with periodontal and bisphosphonate treatment.^57^ To summarize, anti‐osteoclast or antiresorptive drugs is a potential strategy for treating OA through regulating Wnt/β‐catenin signalling.

## 5 Β‐CATENIN SIGNALLING IN OA SYNOVITIS

**FIGURE 1:**
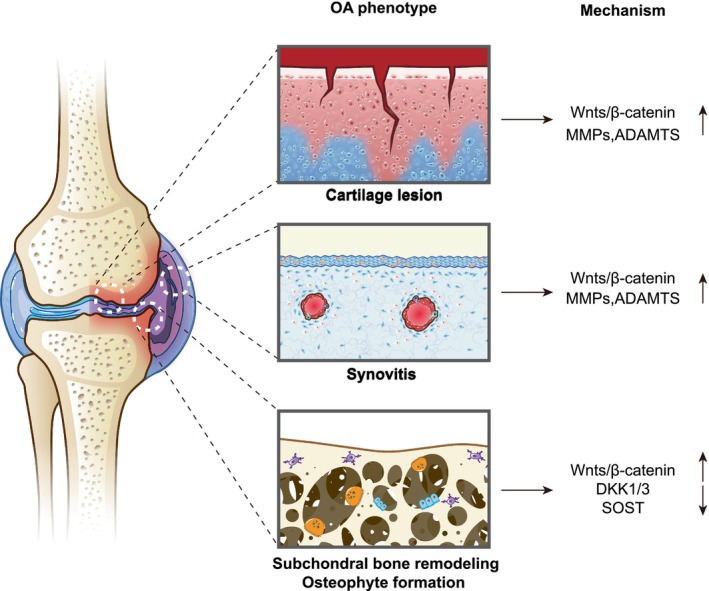
Abnormal β‐catenin signalling in chondrocytes, synovial fibroblast and osteocytes leads to OA‐like structural changes, such as progressive cartilage lesion, synovitis and sclerosis of subchondral bone and osteophyte formation.

It is reported that Wnt/β‐catenin was upregulated in synovial fibroblasts of murine OA and OA‐derived human synovial fibroblasts. Using adenoviral vectors transfecting synovium, overexpression of Wnt8a, Wnt16 and WISP1 all cause the activation of Wnt/β‐catenin in the cartilage, resulted in increased cartilage damage.^58^ This revealed that synovium Wnt signalling crosstalked with cartilage, leading to cartilage degradation. Synovial overexpression of Wnt8a and Wnt16 stimulate MMPs expression, thus aggravating disease progression. Activation of Wnt3a in human OA synovium promote various MMPs expression, whereas inhibiting Wnt signalling decreased synovial‐derived MMPs.^59^ In a study, intra‐articular injection with XAV‐939, a small‐molecule stimulating β‐catenin degradation by stabilizing Axin, were performed to treat mice OA induced by knee destabilization surgery. This study found that XAV‐939 ameliorated OA cartilage degeneration and synovitis through attenuating the proliferation of synovial fibroblasts and type I collagen synthesis in vitro.^60^ Interestingly, another study found that there were distinct functional identities in synovial fibroblasts during post‐traumatic OA in mice, and Rspo2, the Wnt agonist R‐spondin 2, were secreted by Prg4^hi^ lining fibroblasts that may drive pathological interaction between synovial fibroblasts and macrophages as well as chondrocytes after knee injury.^61^ In addition, recent study reported that fibroblast‐like synoviocyte proliferation was inhibited and synovial fibrosis was reduced via regulating Wnt/β‐catenin signalling by low‐intensity pulsed ultrasound treatment.^62^ Thus, Wnt/β‐catenin is a potential target to treat OA synovitis (Figure 1).

## 6 INTERACTION BETWEEN Β‐CATENIN AND OTHER SIGNALLING MOLECULES IN OA

**FIGURE 2:**
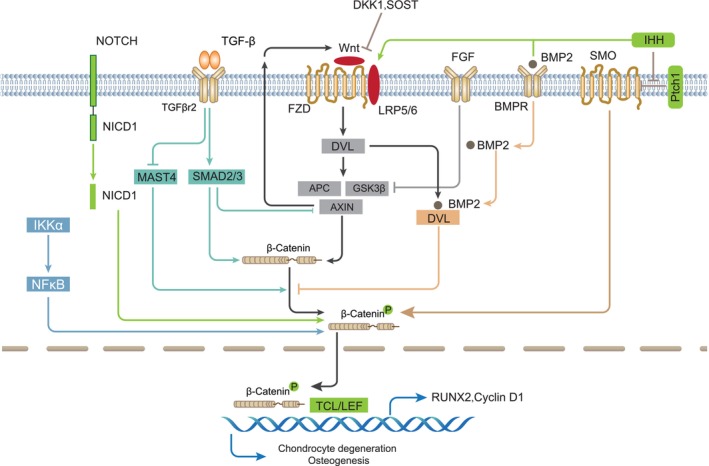
The process of Wnt/β‐catenin signal pathway and interaction between β‐catenin and other signalling molecules in OA. In the activated state, the Wnt ligand activates the Fzd receptor, leading to LRP5/6 phosphorylation, resulting in the formation of the Wnt‐Fzd‐LRP5/6 complex. The binding of the scaffolding protein Dvl to Fzd leads to the dissociation of the Axin‐APC‐GSK‐3β destruction complex as well as the inhibition of β‐catenin phosphorylation by GSK‐3β so that β‐catenin is accumulated in the cytoplasm and transported into the nucleus. In the nucleus, β‐catenin interacts with TCF/LEF family of transcription factors to activate Wnt target genes including Runx2 and cyclin D1. TGF‐β promotes and cooperates with Wnt/β‐catenin in a positive regulatory loop through Smad3 and inhibiting Mast4. Notch can enhance the expression of β‐catenin and its downstream genes, such as cyclin D1 and c‐Myc. NICD inhibits Wnt signalling and the activity of alkaline phosphatase. IKKα can phosphorylate β‐catenin to increase cyclin D1 expression, meanwhile IKKα directly phosphorylates cyclin D1 and suppress cyclin D1 expression. Ihh signalling promotes chondrocyte hypertrophy and osteoblasts differentiation by activating Wnt/β‐catenin signalling. BMP2 inhibits β‐catenin activity through promoting Smad1‐Dvl1 complex formation. Notch inhibits Wnt/β‐catenin signalling at the late differentiation stage in osteocytes. FGF signalling up‐regulates expression of nuclear β‐catenin by inactivating GSK‐3β, whereas Wnt/β‐catenin in turn stimulate FGF23 promoter activity.

Wnt/β‐catenin plays crucial roles in the main pathological changes of OA joint and shares extensive interaction with other signalling pathways participating in OA pathogenesis (Figure 2). These combinatorial signals often bring cell‐type‐ or OA stage‐dependent biological outcomes on cartilage, subchondral bone and synovium that are critical for OA development. The interaction can occur at multiple levels. For example, other signallings can affect the expression and activities of Wnt ligands, receptors, and key moleculars such as Axins, GSK‐3β, Dvl, β‐catenin, and can incorporate into TCF/LEF transcription complexes or induce changes through micro‐RNA, DNA methylation, histone modification to globally control gene expression.

## 7 TGF‐Β/Β‐CATENIN SIGNALLING INTERACTION IN OA

TGF‐β signalling belongs to cytokines of the TGF‐β family with the others members including activins, BMPs, Nodal and growth/differentiation factors (GDFs), which playing essential roles in tissue homeostasis through affecting cell proliferation, differentiation and survival, as well as stem cell renewal.^63^ Three TGF‐β members including TGF‐β1‐β3 were encoded by mammalian genome, which act as homodimers. TGF‐β signalling starts by TGF‐βs that bind at type II or two type I receptors, and subsequent leading to Smad3 and Smad2 phosphorylation.^64^ Followed binding to Smad4, the Smads complexes undergo nucleus translocation and interact with transcription factors to regulate target genes expression.^65^, ^66^

TGF‐β has been shown to be critical for chondrocyte proliferation and extracellular matrix protein synthesis and release.^67^, ^68^ In our previous study, we found that TGF‐β signalling play an essential role in OA pathogenesis.^69^ We have generated chondrocyte‐specific *Tgfbr2* conditional knockout mice (*Tgfbr2* ^*Col2ER*^).^70^ In these mice, tamoxifen‐induced *Tgfbr2* loss is succeed by Cre recombinase driven by the *Col2a1* promoter. Using these mice, we first recapitulate OA initiation and progression similarly found in OA patients. Meanwhile, we found that the *Tgfbr2* gene loss in chondrocytes increased the expression of Col10, Mmp13, Adamts5 and Runx2. Moreover, articular cartilage break‐down, increased mass of subchondral bone and osteophyte formation were found in 3‐month‐old *Tgfbr2* ^*Col2ER*^ mice. Similarly, 6‐month‐old *Tgfbr2* ^Col2ER^ mice exhibit subchondral bone exposure, huge bone mass increase and extensive osteophytes formation. Our results were consistent with the previous findings that reduced expression of TGF‐β receptor in old mice worsens OA progression. To determine if *Tgfbr2* ^Col2ER^ induce OA via regulating *Mmp13* and *Adamts5* expression, we generated ^*Tgfbr2*^/*Mmp13* and *Tgfbr2*/Adamts5 double loss mice. We found that inhibition of Mmp13 expression markedly delayed OA‐like structural changes in *Tgfbr2* ^*Col2ER*^ mice.^71^ However, Adamts5 loss only had weak effect on attenuation of OA progression in *Tgfbr2* ^*Col2ER*^ mice at early time point. Furthermore, *Tgfbr2* ^*Col2ER*^ mice treated with CL82198 (10 mg/kg), a MMP‐13 inhibitor, for 2 months exhibited inhibition of OA development in 3‐month‐old *Tgfbr2* ^*Col2ER*^ mice.^69^ These data indicate that *Mmp13* gene deletion attenuated articular cartilage degeneration. Collectively, loss of TGF‐β signalling in articular chondrocytes causes OA aggravation through up‐regulating *Mmp13* and *Adamts5*.

The cross‐talk between TGF‐β and Wnt/β‐catenin has been studied extensively. The two pathways are intertwined from embryonic development, and molecularly interact at multiple levels, resulting in various changes of associated genes, thereby contributing to OA development.

Our previous study demonstrated that during osteoblast and chondrocyte differentiation TGF‐β cooperates with Wnt in a positive regulatory loop.^72^ In this study, TGF‐β treatment causes a continuous increase expression of β‐catenin in *Axin2* ^*−/−*^ chondrocytes compared to wild‐type chondrocytes. Interestingly, overexpression of Axin leads to strengthened TGF‐β signalling while overexpression of β‐catenin weakens the expression of Smad3‐sensitive reporters induced by TGF‐β. Furthermore, we observe that the inhibition of the Axins is Smad3‐dependent for the absent effect in *Smad3* ^*−/−*^ chondrocytes. Other studies reveal that TGF‐β regulates the expression of Wnts ligands and LRP5 and inhibits the expression of Axin1/2 to activate Wnt/β‐catenin signalling.^72^, ^73^ Recently, study reported that Microtubule Associated Serine/Threonine Kinase Family Member 4 (Mast4) regulates chondro‐osteogenic differentiation of MSCs through activating both TGF‐β and Wnt signal pathway.^74^ TGF‐β1 inhibits the expression of Mast4, enhancing chondrogenesis of MSCs and Mast4 promotes β‐catenin translocated into nuclear and the activity of Runx2, boosting MSCs osteogenesis. Interestingly, in vivo experiment demonstrated that Mast4 deletion in MSCs results in articular cartilage formation and regeneration. These results uncover essential roles of TGF‐β in regulation of β‐catenin through Mast4 in driving MSC differentiating into bone and cartilage. Conversely, Wnt signalling increases Runx2‐mediated expression of TGFβRI independ of β‐catenin to promote TGF‐β signalling. Although Bosch's study demonstrated that canonical Wnts and its downstream protein, WISP1, make TGF‐β shifting from the protective activin‐like kinase 5 receptor (ALK5)‐Smad 2/3 pathway toward the ALK1‐Smad1/5/8 pathway, which induces hypertrophy and terminal differentiation in chondrocytes. It remains to be seen whether this effect is β‐catenin‐independent.^75^ Collectively, the TGF‐β/Smad3‐mediated upregulation of β‐catenin and reduced Axins result in negative feedback loop on TGF‐β signalling and positive feedback loop on β‐catenin signalling.

## 8 BMP/Β‐CATENIN SIGNALLING INTERACTION IN OA

BMPs, first isolated as crucial cytokines for ectopic bone formation, belong to TGF‐β superfamily.^76^, ^77^ The binding of BMP proteins to their receptors, including four type I receptors and three type II receptors, triggers the transduction of BMP signalling. This binding causes phosphorylation of type I receptor by type II receptor. Smad1/5/8 proteins are key mediators in regulating target genes expression in BMP signalling.

BMPs, including BMP2, BMP7 (OP1), BMP4, BMP6 and BMP14 (Growth Differentiation Factor 5, GDF5), play important role in cartilage homeostasis and regeneration.^78^ Previous studies have reported that BMPs have an anabolic effect in vitro, stimulate chondroprogenitors recruitment, and promote synthesis of cartilage collagen and proteoglycan. Compared with conventional soluble BMP2 and BMP7, sustained release of BMP2 and BMP7 using Polyhedrin Delivery System (PODS) strongly induced proliferation in primary chondrocytes and in chondrocyte cell lines. Not surprisingly, both PODS‐BMP2 and PODS‐BMP7 improved cartilage repair and upregulated ECM‐producing genes.^79^ These findings highlight the importance of BMP2 and BMP7 in cartilage homeostasis and point to a protective role for maintaining tissue integrity during OA development. However, the role of BMP4 on chondrocytes has yielded somewhat contradictory results. In a study, BMP4 upregulates proteoglycan synthesis and Col2a1 expression as well as chondrocyte proliferation in bovine chondrocytes and cartilage explants, and similar effects were also observed in human chondrocytes in vitro.^80^, ^81^ Moreover, BMP4 expressed at the osteochondral junction is positively correlated with OA progression, increase the expression of angiogenesis (Angptl4) and osteoclastogenesis (Rankl and Ccl2).^82^ Thus, BMP4 may induce a catabolic and remodelling effect on hypertrophic chondrocytes and osteoblasts. These observations reveal the opposite effect of BMP4 on chondrocytes, hypertrophic chondrocytes, and subchondral bone.

In human chondrocytes, BMP6 promotes proteoglycan synthesis and chondrocyte responses to BMP6 decrease with aging.^83^ It is demonstrated that BMP6 has a pro‐hypertrophic effect on ATDC5 cells.^84^ Similarly, when BMP9 seeded in biodegradable polyglycolic acid scaffolds, chondrocytes underwent ECM mineralization.^85^ BMP9 induce chondrogenesis accompanied by hypertrophy in mesenchymal progenitor cells.^86^ In primary bovine chondrocytes, BMP stimulated hypertrophy remarkably, but this effect was blocked by TGF‐β1‐Smad2/3 signalling.^87^ Therefore, BMP6 and BMP9 signalling possibly has a detrimental effect on cartilage regeneration.

GDF5 is most expressed in joints development and cartilage. Recent GWAS studies have reported that single nucleotide polymorphisms (SNPs) of *GDF5* gene are important risk factors for OA onset in distinct populations.^88^ Therefore, GDF5 represents a vital gene for OA pathogenesis and potential target for OA treatment. However, the precise molecular mechanisms of these SNPs modulating GDF5 in OA pathogenesis remained unknown. It has been reported that GDF5 promotes the synthesis of proteoglycan and expression of COMP but with no effect on COL2A1 synthesis and chondrocyte proliferation.^89^, ^90^ Intra‐articular injections of rhGDF5 halt OA development in the surgical rat OA model in a dose‐dependent manner.^91^ Mechanically, GDF5 reduced MMP13 and increased SOX9 and ACAN expression in human chondrocytes.^92^ Interestingly, GDF5 was also reported to increase α5 integrin subunit, the major receptors of the extracellular matrix, and ACAN, collagen type II and Ihh expression.^90^ Thus, the presence of GDF5, α5 integrin and Ihh protect chondrocytes against hypertrophy. These data suggested that GDF5 play a key role in the maintenance of articular cartilage homeostasis. Targeting GDF5 maybe a candidate therapeutic strategy for OA treatment.

As highly evolutionarily conserved pathways, both BMP and Wnt/β‐catenin play important roles in regulation of osteochondral development, homeostasis and remodelling. They cooperatively or antagonistically regulate similar cellular events depending on cell types and disease developmental stages. A growing evidence indicated that extensive interactions exist between BMP and Wnt/β‐catenin signalling. Our previous work demonstrated that Wnt and BMP interaction have effects on osteoblast differentiation and bone formation.^93^ In this study, both Wnt3a and β‐catenin/TCF4 overexpression increased BMP2 expression while Wnt inhibitors such as sFRP4 and DKK1, ICAT, or FWD1/β‐TrCP, reduced BMP2 transcription. Mechanically, we found that TCF/LEF directly regulate BMP2 transcription.

In the other study, BMP2 increases LRP5 expression and reduces the expression of β‐TrCP, the F‐box E3 ligase degrading β‐catenin and subsequently promotes β‐catenin expression in osteoblasts. Knockout of *β‐catenin* suppresses osteoblast proliferation and differentiation and reduces the responsiveness of osteoblasts to the BMP2 treatment.^94^ Rawadi et al. reported that BMP2 promoted ALP expression depending on Wnt/LRP5 signalling cascade. They also found that a Wnt autocrine/paracrine loop contributes to the effects of BMP2 on extracellular matrix mineralization by osteoblasts.^95^ These findings suggest that BMP2 cooperatively regulate osteoblast function through regulating β‐catenin signalling. Above all, the evidence presented suggest that Wnt/β‐catenin directly regulates BMP2 expression and these two signalling pathways likely reinforce each other in osteoblasts, shedding new lights on the functional crosstalk of these two pathways in differentiation of osteoblast and skeletal homeostasis maintenance.

However, there are conflict data on BMP regulating Wnt signalling in osteoblasts and chondrocytes. Using *Bmpr1a* cKO mice and Wnt reporter *TOPGAL* mice as well as TOPFLASH luciferase, Kamiya et al. found that BMPRIA reduce bone mass and suppress Wnt/β‐catenin signalling through activating *Sost* and *Dkk1* in osteoblasts.^96^ Consistent with this findings, Smad4 was found to competitively bind β‐catenin and inhibit the latter binding with TCF/LEF transcription complex. Thus, deletion of Smad4 boosts bone formation through regulating Wnt/β‐catenin signalling either in osteoblast or in the crest neuron stem cell.^97^ Similar mechanism with Smad4, BMP2 inhibits β‐catenin activity through promoting Smad1‐Dvl1 complex formation. These results provide new perspective by which BMP negatively regulates Wnt/β‐catenin to reduce bone mass in osteoblasts. In OA cartilage, Papathanasiou and colleagues proposed that BMP2 interacts with canonical Wnt/β‐catenin in controlling chondrocyte hypertrophy and the synthesis of MMPs and ADAMTS. Interestingly, they found that both BMP2 and BMP4 were produced by human chondrocytes in end‐stage OA. However, BMP2, but not BMP4, upregulated expression of LRP5, the key co‐receptor in Wnt/β‐catenin signalling.^98^ This is not consistent with the effect of BMPs in osteoblasts.

## 9 NOTCH/Β‐CATENIN SIGNALLING INTERACTION IN OA

Notch signalling is an evolutionarily conservative pathway during development and homeostasis, which is involved in cellular communication, cell fate decisions, pattern regulation, differentiation.^99^, ^100^ The Notch pathway mediates intercellular communications, in which signal sending and receiving cells interacted by ligand receptor binding between two neighbouring cells.^101^ Ligands, receptors and downstream effectors are three major components of the Notch signalling. The ligands for binding Notch receptors include Jagged 1,2 and Dll 1, 3, 4, belonging to Delta/Serrate/Lag2 (DSL) family. In mammals, Notch receptors have been identified, containing both extracellular and intracellular domains. Notch signalling initiates by a Notch receptor protein binding of a ligand from one cell to the extracellular domain of receptor of the other cell, or ligand‐independent receptors. Following activation, the receptors' extracellular subunits were dissociated from its transmembrane subunits, and sequentially cleaved by ADAM family of metalloproteases complex and γ‐secretase, resulting in the release of activated Notch intracellular domain.^102^ In the canonical Notch pathway, NICD enters the nucleus and forms RBP‐J (recombination signal binding protein for immunoglobulin kappa J region), along with the coactivator Mastermind‐like (MAML) complex, and eventually activates the expression of downstream effectors, such as Hes family proteins. In the non‐canonical Notch pathway, RBP‐J‐independent non‐canonical downstream effectors such as Iκκ, NF‐κB, Myc, p21 and PI3K/AKT are activated.

Notch has been proved to be critical molecular signalling in regulating cartilage development and joint homeostasis. Previous study reported that knockout of *RBPjκ* in mice limbs (*Prx1Cre;Rbpjκ* ^*fl/fl*^) causes early, severe and progressive OA‐like pathology.^103^ Additionally, haploinsufficiency of *Rbpjκ‐*floxed alleles (*Prx1Cre;Rbpjκ* ^*fl/wt*^) results in progressive cartilage degeneration. The results indicated that canonical Notch signalling is essential for cartilage homeostasis. To study the role of the core Notch signalling component, RBPjκ in joint chondrocytes and subchondral osteoblasts, Liu et al. found that deletion of *RBPjκ* from articular chondrocytes, but not subchondral osteoblasts, results in degenerative OA‐like pathology through recruiting fibrotic cells that produce cartilage‐degenerative enzymes.^104^ Therefore, Notch signalling may not play a major role in osteoblast‐lineage cells. In a study, sustained Notch activation and transient Notch activation mouse models were generated to overexpress the Notch1 intracellular domain (NICD) in cartilage. They observed progressive OA‐like pathology in sustained Notch activation mouse but increased expression of cartilage matrix protein and protected joint structure in transient *Notch* activation mouse. Mechanically, the cartilage degradation, fibrosis, and OA development caused by Notch activation may be through the activator of transcription 3 (STAT3) and interleukin‐6‐signal transducer as well as mitogen‐activated protein kinase signalling pathways.^105^

Interactions between Notch and Wnt/β‐catenin have long been found in cancer. It has been reported that activated Notch can enhance the expression of β‐catenin and its target genes like cyclin D1, c‐Myc, which in turn promote bidirectional signal transmission.^106^ In this context, Notch/Hes1 signalling lies in upstream of β‐catenin and TCF.^107^ However, the research concerned the interactions between Notch and Wnt in OA is less. Though notch signalling may not play a major role in osteoblast‐lineage cells, the interplay between Notch and Wnt in OA cartilage could refer to that in osteoblast‐lineage cells. In osteoblast precursor cells, hypoxia suppressed cell proliferation and upregulation of activated β‐catenin and its target genes, whereas Notch repression increased cell proliferation in a concentration‐dependent manner. This study presented the contradictory results between Notch and Wnt under hypoxia and that Wnt‐Notch signal interaction orchestrate osteoblast proliferation.^108^ A study demonstrated that reduced proliferation and osteogenic differentiation but enhanced adipogenic differentiation were observed in tuberous sclerosis 1 (Tsc1)‐loss bone marrow stromal cells. Mechanistically, TSC1 loss inhibits autophagy and upregulate Notch1 expression through triggering GSK‐3β‐independent β‐catenin degradation.^109^ To understand the mechanisms of overexpression of NICD impairing osteoblastogenesis, reduced Wnt3a, cytoplasmic expression of β‐catenin and Wnt downstream gene were observed in Notch overexpressed ST‐2 cells.^110^ NICD inhibits Wnt signalling and the activity of alkaline phosphatase. However, this effect was partially blocked by β‐catenin mutation, or using a GSK‐3β inhibitor to stabilize β‐catenin. Therefore, overexpressed NICD suppress Wnt signalling.^111^ It is well‐known that osteocytes are originated from osteoblasts and comprise the most of bone cells. To explore how Notch functions in the process of osteoblasts differentiating into osteocytes, Shao et al. study demonstrated that Notch inhibited Wnt signalling and β‐catenin translocation into nucleus at the late differentiation stage of osteocytes.^112^ These data provide new insights of Notch signalling in osteocyte differentiation.

## 10 IHH/Β‐CATENIN SIGNALLING INTERACTION IN OA

The essential role of Hedgehog signalling in cell proliferation and apoptosis, tumorigenesis and development, stem cells differentiation and ciliary movement were well documented.^113^ The Hedgehog family members comprise three vertebrate homologues, including Indian hedgehog (Ihh), sonic hedgehog (Shh) and desert hedgehog (Dhh). Ihh is necessary for skeletal development, particularly for endochondral bone formation, whereas Shh plays important role for neuronal development and Dhh for gonads. The main components of this pathway include hedgehog ligands, smoothened (Smo) and patched1 (Ptch1) transmembrane proteins, SUFU (Suppressor of Fused), COS2 (Costal 2) and inhibitors such as protein kinase A (PKA) and supernumerary limbs (SLIMB) in the cytosol, and the transcription factors glioma‐associated oncogene homologue 1 (Gli). In the absence of Hh ligand, the receptor Ptch inhibits the co‐receptor Smo, and Gli binds to SUFU and is processed into GliR, which inhibits the transcription of target genes. When Hh binds to Ptch1, relieving its inhibition on Smo, Gli is dissociated from SUFU and shuttles to nucleus, ultimately inducing the expression of target genes, such as Ptch1, Gli1, and Hip.^114^ In the non‐canonical Hh signalling pathway, activated Gli increases target genes expression through Phospholipase Cγ (PLC‐γ)‐inositol 3‐phosphate (IP3)‐diacylglycerol (DAG) and IGF2 signalling.

In 2009, a study reported that activation of hedgehog induces OA and that Runx2 potentially mediates OA development by regulating Adamts5 expression.^115^ The subsequent studies identified Ihh signalling plays an important role in OA pathogenesis and block of Ihh were therapeutic strategies to inhibit OA cartilage degradation.^116^, ^117^ Zhou et al. reported that deletion of *Ihh* in cartilage using a conditional deletion construct (*Col2a1‐Cre* ^*ERT2*^ *;Ihh* ^*fl/fl*^) exhibits significantly more cartilage damage.^118^ A study discovered a rare missense mutation of SMO gene (NP_005622.1:p. Arg173Cys) strongly linked with hip OA.^119^ The SMO gene mutation changes the amino acid arginine at site 173 with cysteine. It remains to be clarified whether the mutation of SMO‐R173C indeed activate Hh signalling. Further study is needed to analyse OA phenotype of *SMO‐R173C* mutant mice and determine whether this mutation results in activation of Ihh signalling.

Wnt/β‐catenin and Ihh signallings are crucial in regulating chondrocyte differentiation, limb development and articular cartilage homeostasis. These two pathways interact to control osteoblast differentiation, the fate of chondrocyte and synovial joint formation. In a study, upregulated Ihh signalling in the cartilage promotes chondrocyte hypertrophy by activating Wnt/β‐catenin signalling.^120^ Another study demonstrated that Prrx1+ mesenchymal stem cells specific loss of *Ptch1* promotes chondrogenesis and osteogenesis but suppresses adipogenesis. *Ptch1* deficiency causes OA‐like phenotypes in Smo‐Gli1/2‐dependent manners. As a negative regulator and a target gene of Ihh signalling, *Ptch1* loss increases Wnt5a/6 and β‐catenin expression. These findings suggest that β‐catenin is the downstream regulator of Ihh signalling in chondrocytes differentiation and hypertrophy.^121^ Similar in osteoblast, β‐catenin is indispensable for Ihh signal transduction and the expression of osterix during osteoblast differentiation. However, others reported that β‐catenin could regulate Ihh signalling to inhibit chondrocyte apoptosis.^122^

## 11 NF‐ΚB/Β‐CATENIN SIGNALLING INTERACTION IN OA

The members of NF‐κB family compose of NF‐κB1 p50/p105, NF‐κB2 p52/p100, RelA/p65, c‐Rel and RelB. These proteins act as dimers to κB sites in promoters and enhancers of downstream genes to regulate transcription. The NF‐κB signalling plays crucial role in stress responses, innate and adaptive immunity, inflammation and lymphatic organogenesis.^123^, ^124^ In the classical NF‐κB signalling, NF‐κB/RelA protein binds to inhibitory I‐κB protein. Pro‐inflammatory cytokines, antigen receptors and growth factors activate an IKK complex (I‐kβ, Ikkα, and NEMO). The activation of IKK complex are involved in NF‐κB activation and in mediating important biological functions, which mediated by the phosphorylation of IKK kinase or by autophosphorylation. I‐κB Phosphorylation induces itself ubiquitination and proteasome degradation, setting free the NF‐κB p65 complex. After phosphorylation, acetylation, or glycosylation, the active NF‐κB p65 complex went into nucleus translocation, inducing target gene expression including AP‐1, Stat, Ets, etc. In the alternative NF‐κB pathway, the NF‐κB2 p100/RelB complex is the key regulator and inactivated in the cytoplasm. Signal transduction activates the kinase NIK via lots of receptors, including BR3, Lt‐βr and CD40, which in turn activates the IKKα complex and phosphorylates NF‐κB. Phosphorylation of NF‐κB2 p100 results in its own ubiquitination and then processed by the proteasome to NF‐κB2 p52. In this way, a transcriptional NF‐κB p52/RelB complex can be generated and transported into the nucleus to induce cascades.

The interplay between Wnt/β‐catenin and NF‐κB signalling has long been discovered. As a bottleneck in classical Wnt signalling, GSK‐3β is involved in regulating the activation of Wnt/β‐catenin signalling, which mediated β‐catenin phosphorylation and its proteasomal degradation. However, IKKα can phosphorylate β‐catenin at different phosphorylation sites targeted by GSK‐3β and stabilizes β‐catenin, leading to TCF‐dependent expression of cyclin D1.^125^, ^126^, ^127^ A well‐documented mechanism of Wnt/β‐catenin regulation by NF‐κB signalling is through cyclin D1, a well‐known target gene of Wnt/β‐catenin. Previous study showed conflicting results about IKKα regulating cyclin D1 expression. For instance, oestrogen receptor and SRC‐3 coactivator mediated cyclin D1 expression require IKKα‐dependent transcription complex.^128^ It is reported that IKKα directly phosphorylates cyclin D1 at Thr286 and IKKα suppressing cyclin D1 expression by reducing Erk1/Erk2‐dependent transcription.^129^, ^130^ IKKα‐induced phosphorylation of cyclin D1 is involved in cell proliferation and apoptosis.^131^ These data suggest a complex, context‐dependent communications between Wnt/β‐catenin and NF‐κB that clearly needs further investigation. More recently, using lentivirus‐mediated *cyclin D1* knockdown or overexpression, Chen et al. found that cyclin D1 upregulates Wnt3/β‐catenin signalling to promote chondrocyte proliferation and inhibit chondrocytes apoptosis.^33^ Wnt/β‐catenin not only reduced basal level expression of MMP1, MMP3, and MMP13 but also IL‐1β‐induced upregulation of MMPs via inhibiting NF‐κB in human chondrocytes.^132^ This study indicates that Wnt/β‐catenin counteracted NF‐κB signalling and had a protective role on human chondrocytes. Although AnxA6 is not good for human articular chondrocytes, which have been tested to affect Wnt/β‐catenin and NF‐κB interaction in membrane by repressing β‐catenin and boosting NF‐κB activity. Searching for novel molecules that targeting Wnt/β‐catenin and NF‐κB signalling interaction may be promising strategy for OA treatment.

## 12 FGF/Β‐CATENIN SIGNALLING INTERACTION IN OA

FGF proteins exert their biological effects by binding tyrosine kinase receptors, FGFR1‐4, which involved in regulating cell fate, angiogenesis, immunity, and metabolism.^133^ Currently, there were 22 FGF ligands identified. Among them, the Fgf15/19 (FGF19, −21, −23) subfamily members encode endocrine FGF ligands that bind to Klotho family proteins and activate FGFRs.^134^ The Fgf11 (FGF11, FGF12, FGF13, FGF14) subfamily genes encode intracellular FGFs, which interact with voltage gated sodium (Nav) channels. The remaining FGF subfamily genes encode paracrine FGF ligands interacting with FGFR and sheparin‐sulfate proteoglycans.

The malfunction of FGF signalling axis causes different diseases, such as chondrodysplasia, chronic kidney disease, dwarfism syndromes and various tumours.^135^ This signalling initiates by the binding of FGFs to their receptors, which stimulates the conformational changes of FGFRs and activation. Activated FGFRs phosphorylate FGFR substrate 2α, which binds to growth factor receptor‐bound 2 (Grb2). Grb2 will subsequently bind to son of sevenless (SOS), GRB2 associated binding protein 1 (GAB1), and Cbl through activating Ras/Raf/MAPKs, including JNK‐MAPK, ERK‐MAPK and p38‐MAPK.^136^ Simultaneously FGFRs also activate PI3K, STAT and phosphorylate protein kinase C γ (PLCγ). Several molecules regulating FGF signalling were discovered including Sprouty (Spry), SEF, MKP, XFLRT, which belong to FGF synexpression group and are tightly co‐expressed with FGFs.

FGF/FGFR signalling plays an important role in bone and cartilage development and osteoarthritis. Previous studies demonstrated that FGF1, FGF2, FGF7, FGF8, FGF9, FGF18 and FGF23 are associated with OA pathogenesis. It has been reported that FGF23 increased in human OA chondrocytes, and meanwhile exogenous expression of FGF23 promotes chondrocytes terminal differentiation and increases MMP13 and FGFR1 production in chondrocytes.^137^, ^138^, ^139^ FGF signalling has been showed to upregulate expression of nuclear β‐catenin by inactivating GSK‐3β whereas Wnt/β‐catenin in turn was reported to stimulate FGF23 transcriptional activity in osteoblasts.^134^, ^140^ These studies suggest that FGF23 and canonical Wnt/β‐catenin signalling cooperatively contributes to OA development. A study reported that Axin1 inhibits Raf1‐MEK‐ERK1/2 cascade via a β‐catenin‐dependent manner in fibroblasts, indicating that Wnt/β‐catenin may interact with FGF signalling during synovitis and chondrogenesis. In addition, Wnt signalling enhances FGF‐mediated inhibition of chondrocyte differentiation, whereas FGFs stimulates ERK‐mediated Wnt/β‐catenin signalling activation.^141^, ^142^ Furthermore, double activation of FGF and Wnt/β‐catenin signallings results in increased the expression of MMPs and Adamts as well as the subsequent loss of cartilage extracellular matrix.^142^ Previously using *Axin1* ^*Agc1ER*^ mice in which deleting *Axin1* in aggrecan‐expressing chondrocytes, we found that expression of *Fgfr1*, *Fgfr2*, and *Fgfr3* as well as FGF downstream molecule pERK1/2 were upregulated in condylar cartilage of *Axin1* KO mice.^143^ These results indicate that loss of *Axin1* in condylar chondrocytes causes OA‐like change in temporomandibular joint through regulating β‐catenin and FGF signalling. Together, these findings imply that FGF signalling and Wnt/β‐catenin cooperatively worsen OA progression and simultaneously inhibiting both signalling pathways may be new strategy to treat OA.

## 13 RUNX2/Β‐CATENIN SIGNALLING INTERACTION IN OA

As an extremely important transcription factor, Runx2 plays a major role in regulating osteoblast and chondrocyte differentiation.^15^ It has been reported that Runx2 induces expression of hypertrophy‐related genes, such as *Mmp13*, *Adamts5* and *Col10* and promotes chondrocyte hypertrophy and ossification.^144^ *Runx2* heterozygous knockout mice and chondrocyte‐specific *Runx2* knockout mice exhibit reduced OA development, and decreased expression of MMP13.^145^, ^146^, ^147^ Additionally, Runx2 overexpression in chondrocytes led to post‐traumatic OA progression.^148^ Runx2 is also essential for the proliferation and differentiation of osteoprogenitors. Previous studies have implicated that Ihh is required for Runx2 expression in endochondral bone, and Runx2 can induce multipotent mesenchymal cells differentiating into preosteoblasts.^149^ Moreover, it is reported that Runx2 boosts osteoblast progenitors cells proliferation through increasing Fgfr2 and Fgfr3.

Interestingly, Runx2 promotes suture mesenchymal cells proliferation and the osteogenic differentiation of mesenchymal cells through directly regulating Wnt signalling.^150^ These results suggested that Runx2 directly promotes Wnt/β‐catenin. In embryonic stem cells and osteoblasts, β‐catenin forms a complex with Smad4 (or Smad1/5), which acts on the TCF/LEF and Smad binding sites of the Msx2 promoter regions, regulating Msx2 transcription after entering the nucleus.^151^ Additionally, Msx2 interacts with Msx2‐binding sites on the Runx2 promoter to regulate the Runx2 transcription.^152^ It has been reported that β‐catenin can directly act on the TCF binding site of the Runx2 promoter to upregulate the Runx2 in rats aortic smooth muscle cells.^153^ Meanwhile, aberrant activation of β‐catenin mediated Runx2 resulted in osteoblast and chondrocyte dysfunction, joint degeneration, and bone mass increase. Importantly, increased Runx2 expression through β‐catenin is required for osteoblasts differentiation and the suppression of the chondrogenic differentiation.^154^, ^155^ Since studies have reported that β‐catenin binds to the *Runx2* promoter and promotes its expression,^154^, ^155^, ^156^ the interaction between Runx2 and β‐catenin lies in that Runx2 appears to serve as direct downstream target of β‐catenin and it directly or indirectly boosts Wnt/β‐catenin in a positive feedback loop in chondrocytes during endochondral bone development.

## 14 CONCLUSIONS

OA is a common chronic disease affecting articular tissues of the whole joints. Understanding the molecular mechanisms of OA pathogenesis could help us to find novel approaches to slow or even to revert OA pathological changes. Pathological mechanisms of OA are complicated and involve varieties of factors. Considering large number of identified important molecules, understanding of the key signalling pathway and the interactions between them represents a huge challenge in OA research. Based on the information described here, a large number of evidence highlights the major role of Wnt/β‐catenin in OA pathogenesis. Wnt/β‐catenin signalling is very likely the dominant one among these pathways in OA occurrence and development. Future research may need to consider identifying whether administration of drugs targeting the Wnt/β‐catenin signalling and combining with new drug delivering technology to alleviate OA disease phenotype.

Despite our knowledge on OA pathogenesis evolved greatly, the aetiology and molecular mechanisms of OA remain unclear. As discussed in this review, TGF‐β, BMP, Notch, Ihh, NF‐κB, FGF, Runx2 signalling pathways are important participants in OA pathogenesis and their cross‐talk with Wnt pathways greatly affect OA development. TGF‐β promotes and cooperates with Wnt/β‐catenin in a positive regulatory loop on Wnt/β‐catenin during osteoblast and chondrocyte differentiation. Additionally, TGF‐β regulates β‐catenin through Mast4 in determining MSC development. Wnt/β‐catenin regulates BMP2 expression and these two signalling pathways likely reinforce each other in osteoblasts, whereas conflicting results on BMP regulating Wnt signalling in osteoblasts and chondrocytes were necessary to be elucidated. β‐catenin is the downstream regulator of Ihh signalling in chondrocytes differentiation and hypertrophy. However, β‐catenin regulates Ihh signalling to inhibit chondrocyte apoptosis. IKKα can phosphorylate β‐catenin to increase cyclin D1 expression, meanwhile IKKα directly phosphorylates cyclin D1 and suppress cyclin D1 expression. Wnt/β‐catenin cooperates with FGF signalling to worse OA progression. Runx2 appears to not only be direct downstream target of β‐catenin but also directly or indirectly boost Wnt/β‐catenin in a positive feedback loop in chondrocytes and endochondral bone development. Although the way of Wnt/β‐catenin interacts with the other signalling molecules remains to be elucidated, a precise clarification on the contribution of Wnt/β‐catenin signalling and the interaction network could provide potential targets for the treatment of OA. Further studies using double or triple knockdown or knock‐in strategies to determine functional roles of their interaction with Wnt/β‐catenin were required for uncovering the nature of these events.

The Wnt/β‐catenin pathway contributes to inflammatory and neuropathic pain through both central and peripheral mechanisms, which involved in the activation of macrophages and the expression of pro‐inflammatory cytokines and chemokines. However, the understanding of Wnt/β‐catenin signalling in the molecular mechanisms of OA pain is limited. In one of our recent studies, we found, for the first time, that β‐catenin interacts with TCF7 in chondrocytes and directly regulates the expression of CCL2. CCL2 have been previously shown to mediate OA‐related pain.^157^ Targeting Wnt/β‐catenin pathway may be a promising therapeutic intervention for OA pain. Further investigations to determine how Wnt/β‐catenin signalling regulates other pain‐related molecules and signalling pathways, such as inflammatory cytokines, chemokines and molecules important for nerve and immune functions.

Furthermore, consequences of uncontrolled systemic dysregulation of Wnt/β‐catenin signalling in the body or the whole joint might not be good choice for the treatment OA. In view of the importance of Wnt/β‐catenin signalling in cancers, Wnt inhibitors have been developed as new drugs for cancer patients with high Wnt signalling. Orally administered PORCN inhibitors, which suppress the secretion of Wnt ligands have been examined with side effects such as loss of bone mass and bone resorption increase.^158^ The side effects of FZD antagonists/monoclonal antibodies, such as Vantictumab and ipafricept include constipation, vomiting, tiredness, diarrhoea, abdominal pain and bone metabolism disorder.^159^, ^160^ Inhibiting β‐catenin or its target genes could induce fatigue, diarrhoea, nausea, anorexia, thrombocytopenia, reversible elevated bilirubin and hypophosphatemia.^161^, ^162^ It seems that precise targeting of the important molecule in the Wnt/β‐catenin signalling, rather than global inhibition, is an optimized resolution to develop more effective and less side effect of anti‐OA drugs. Identification of these new targets working on the Wnt signalling interactive network requires the understanding of detailed molecular mechanisms.

## AUTHOR CONTRIBUTIONS

Conceptualization, preparation and revision of the manuscript, D.C., H.Y., L.T., K.L.; methodology, J.F., Q.Z., W.L.; manuscript preparation, J.F., Q.Z., F.P., Z.Z. All authors have read and agreed to the published version of the manuscript.

## CONFLICT OF INTEREST STATEMENT

The authors declare no conflict of interest.
